# Supplementary material for: Venomix: a simple bioinformatic pipeline for identifying and characterizing toxin gene candidates from transcriptomic data
Source: PeerJ. 2018 Jul 31;6:e5361. doi: 10.7717/peerj.5361 (PMC6074769; doi:10.7717/peerj.5361)
Supplement: Supplemental Information 3 [file peerj-06-5361-s003.gz › FinalOutput_E-20/Snaclec_aspercetin_subunit_alpha_2/finaltree.pdf]

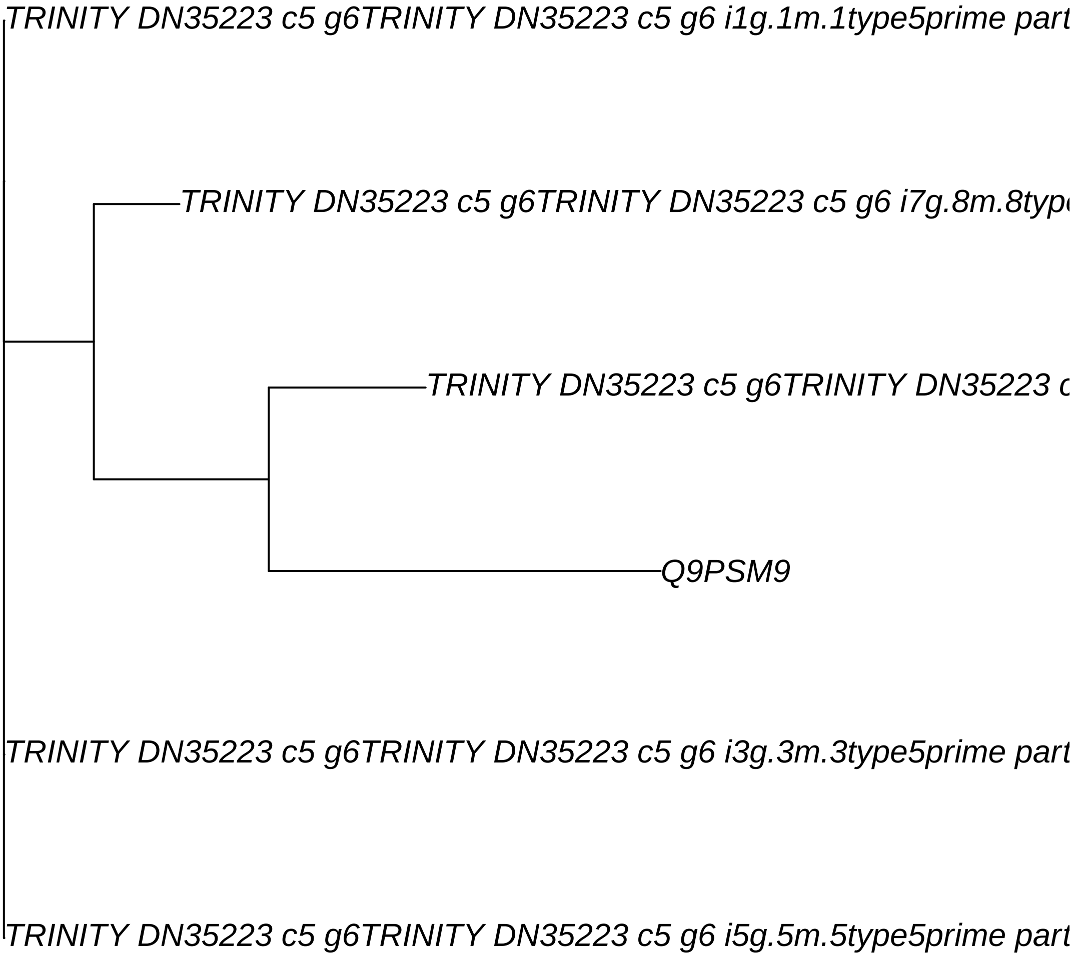
TRINITY DN35223 c5 g6TRINITY DN35223 c5 g6 i1g.1m.1type5prime part
TRINITY DN35223 c5 g6TRINITY DN35223 c5 g6 i7g.8m.8typ
TRINITY DN35223 c5 g6TRINITY DN35223 c
Q9PSM9
TRINITY DN35223 c5 g6TRINITY DN35223 c5 g6 i3g.3m.3type5prime part
TRINITY DN35223 c5 g6TRINITY DN35223 c5 g6 i5g.5m.5type5prime part
